# Reverse wedge effect following intramedullary nailing of a basicervical trochanteric fracture variant combined with a mechanically compromised greater trochanter

**DOI:** 10.1186/s12891-020-03212-6

**Published:** 2020-03-28

**Authors:** Yu Zhang, Jun Hu, Xiang Li, Xiaodong Qin

**Affiliations:** Department of Trauma, the First Affiliating Hospital of Nanjing Medical University & Jiangsu Province Hospital, 300 Guangzhou Road, Nanjing, 210029 China

**Keywords:** Trochanteric fracture, Intramedullary nailing, Intraoperative complication

## Abstract

**Background:**

To introduce an unreported intraoperative complication in intramedullary nailing (IN) of an anatomically reduced trochanteric fracture variant characterized by a basicervical fracture line and coronally disrupted greater trochanter (GT).

**Methods:**

A total of 414 trochanteric fractures (TF) treated with intramedullary nails from 2013 to 2017 were included in this study. After analysis of intraoperative fluoroscopy data, 33 cases, including 21 females and 12 males, with a mean age of 72.5 years (33 to 96 years) were identified for internal rotation of the cephalocervical fragment and inferior opening at the basicervical fracture line caused by nailing a satisfactorily reduced TF. The morphological features of this group of patients were analyzed on computed tomography (CT) scan. On radiograph, the magnitude of the displacement and final femoral neck-shaft angle (NSA) were measured.

**Results:**

CT analysis demonstrated that the basicervical fracture line and the posterolateral fragment (PLF) detached from the GT were the two dominant features of this cohort. They were classified according to the number of main fragments: a 3-fragmentary subgroup containing three consistent fragments (cephalocervical fragment, PLF and distal femoral shaft) and a 4-fragmentary subgroup embracing one additional fragment (lesser trochanter). The four subtypes were as follows: the 3-fragmentary S indicating a small PLF (6 cases), the 3-fragmentary M presenting a moderate PLF (3 cases), the 3-fragmentary L standing for the PLF involving whole lesser trochanter (LT) (4 cases) and the 4-fragmentary GL incorporating separated PLF and LT fragments (20 cases). Geological analysis demonstrated that the majority of the basicervical fracture lines (81.8%) just crossed the center of the piriformis fossa, while the others marginally involved the medial wall of the GT. Postoperatively, the mean width of the inferior opening at the basicervical region was 9.2 ± 4.6 mm. The mean NSA was 135.2 ± 7.8 degrees. The comparison between the 3- and 4-fragmentary subgroups revealed no significant differences in magnitude of displacement and NSA.

**Conclusion:**

This unreported intraoperative complication predominantly occurred in the intramedullary nailed basicervical trochanteric fracture variant combined with a PLF from the GT. The magnitude of the secondary displacement was substantial and resulted in a relative valgus reduction. This secondary displacement was caused by an impingement of the reamer with the superolateral cortex of the cephalocervical fragment and should be addressed during the operation.

**Level of evidence:**

Therapy IV.

## Background

The increase in the aging population worldwide has resulted in a growing amount of TF among geriatric people, which presents challenges to the public health system and to orthopedic trauma surgeons [1]. Surgical intervention has been the preferred treatment for TF patients because of the benefits of effective pain control and the opportunity for early weight bearing. Compared to dynamic hip screw plates, INs are gaining more popularity and could provide superior stability to unstable TF patterns that are characterized by posteromedial comminution, reverse oblique configuration, lateral wall disruption, GT disruption and basicervical variants [2, 3].

**Fig. 1:**
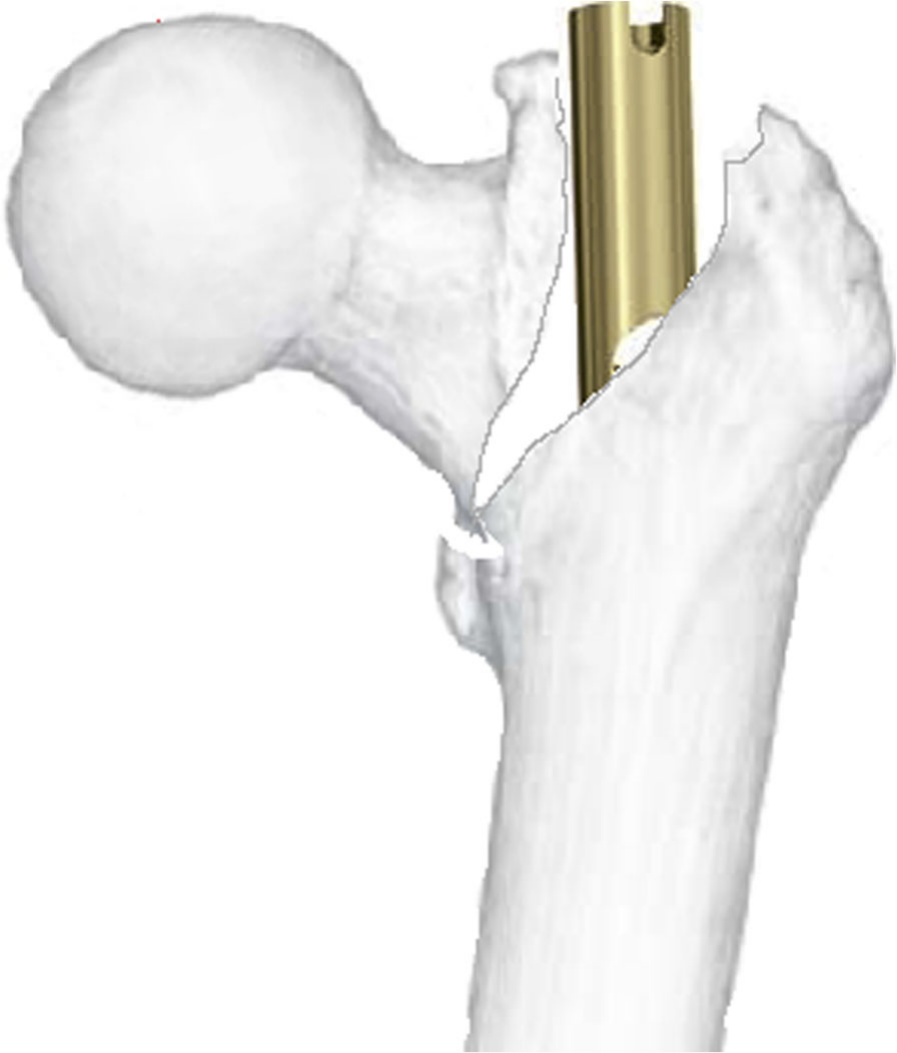
The simulated diagram depicting the “wedge effect”, where the intramedullary nail insertion causes lateralization of the femoral shaft and varus malalignment

At the same time, much attention has been paid to the increasing postoperative complications associated with the IN application. Those complications include sliding screw cut-out or cut-through [4], extensive fracture collapse and reduction loss, thigh pain associated with nail tip collision, and varus malunion. However, the intraoperative complications are less noticeable. Recently, Hak reported an uncommon intraoperative complication when IN was performed to stabilize a group of TFs: a varus malreduction presented as a secondary fracture displacement caused by inserting a cephalated IN from the tip of the GT [5]. It was featured by a lateral displacement of the femoral shaft and an opening of the superior part of the primary fracture line (trochanteric region), which was later named the “wedge effect” by O’Malley [6] (Fig. 1).

**Fig. 2:**
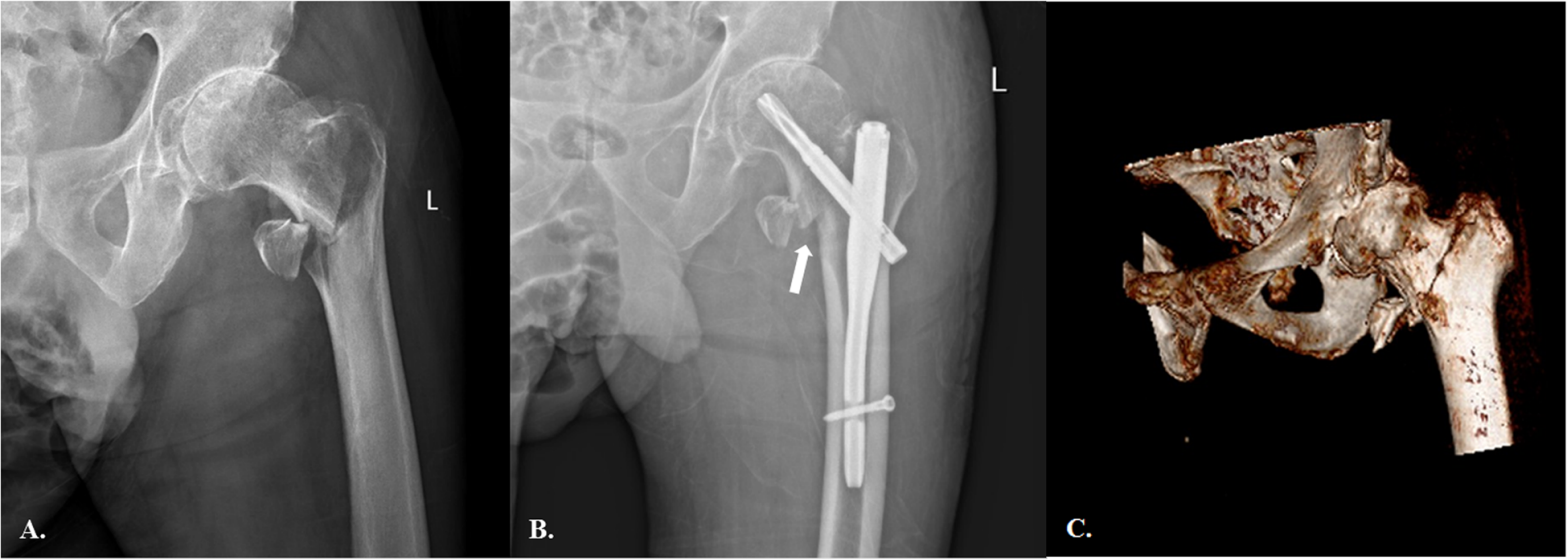
**a** The preoperative anteroposterior (AP) radiograph showing an apparently simple AO-31A1.3 (2018 version) pertrochanteric fracture. **b** Following fixation with PFNA, there was an obvious inferior gap at the basicervical region (white arrow) and moderate valgus deformity. The lucency around helix indicates a micro-motion and instability of the fracture-implantation complex. **c** However, the preoperative 3D-CT revealed that this case was a basicervical trochanteric fracture variant. On preoperative radiograph, the external rotation of the femoral shaft made the fracture line assessment and classification incorrectly

Interestingly, we also noticed another pattern of intraoperative complications closely associated with inserting a trochanter tip starting point IN in a cohort of TFs (Fig. 2). In contrast to the aforementioned “wedge effect”, the reaming/IN insertion generated internal rotation of the cephalocervical fragment and an inferiorly oriented gap at the primary fracture line (basicervical region). This pattern was named the “reverse wedge effect”. Further studies revealed that this intraoperative complication completely occurred in an uncommon TF pattern distinguished by the primary fracture line at the basicervical region (basicervical TF variant) and a detached PLF from the GT. The purpose of the present study was to investigate the incidence of the “reverse wedge effect” at a level-one tertiary trauma center, summarize its morphological features and analyze its cause and potential influence on treatment outcomes.

## Methods

We obtained Institutional Review Board approval for this study. A total of 414 TF cases that underwent IN fixation at our hospital between January 2013 and January 2017 were included in this study. The inclusion criteria were skeletally mature, unilateral TF with complete radiographs, intraoperative fluoroscopy and preoperative CT scan which improved our understanding of the fracture characteristics and the degree of instability. The exclusion criteria were as follows: (1) patients younger than 18 years; (2) high-energy injury or multiple injuries such as car accidents or high-altitude fall injuries; and (3) patients with pathological fractures and previous histories of hip malformation or surgery of either hip. By observing intraoperative fluoroscopy, the TF cases demonstrating the “reverse wedge effect” during reaming or nail insertion were identified.

The demographic information of this cohort was extracted from patient medical records. The radiographic data were available in the Picture Archiving and Communication System (PACS), and the measurements were carried out with the built-in gauge tools. One of the senior surgeons at the hospital classified the fractures according to the AO/OTA classification (2018 version) on plain radiographs (H.J.).

**Fig. 3:**
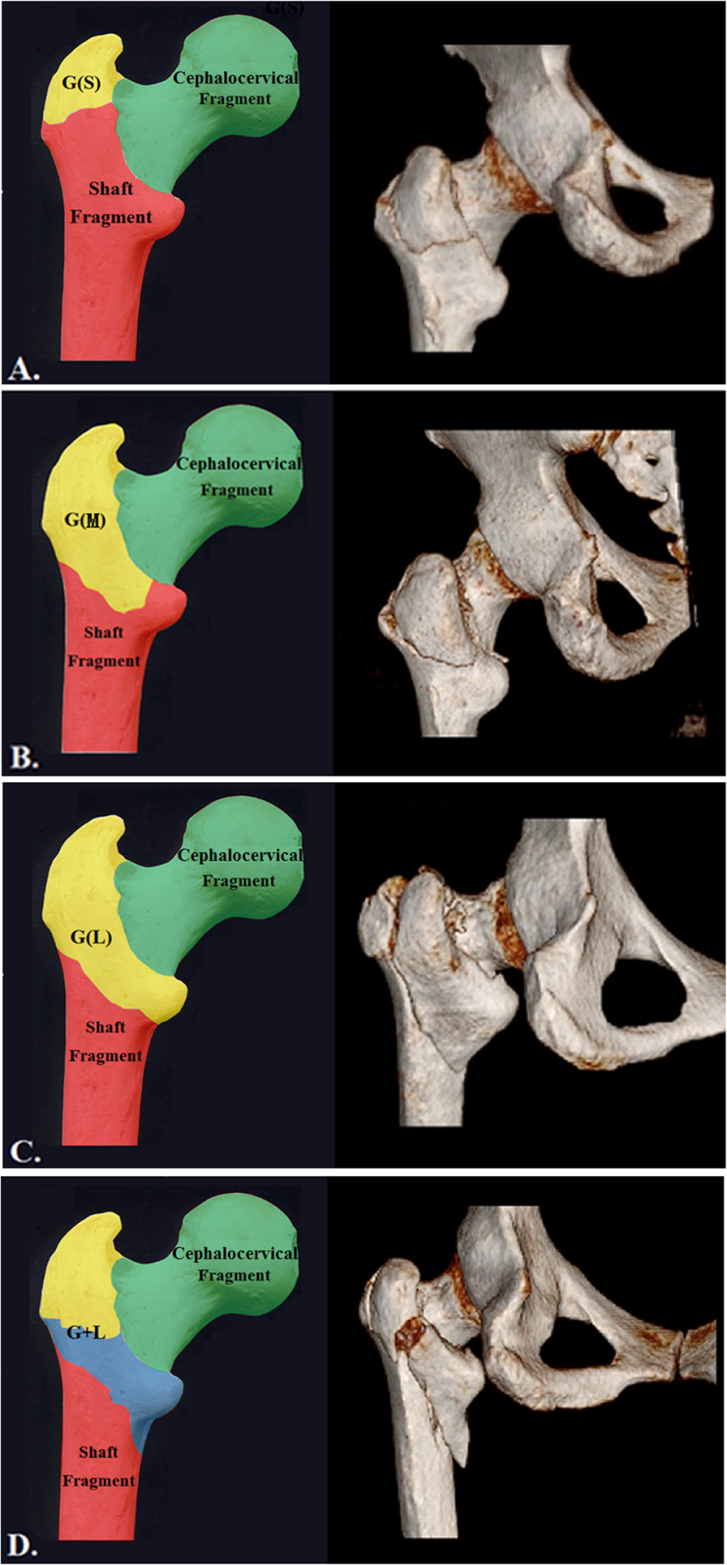
Based on the number of fragments, the fractures were divided into four subtypes. The cephalocervical segment and shaft segment were two consistent fragments. **a** The PLF in the subtype3-fragmentary S is relatively small. **b** In the subtype 3-fragmentary M, the PLF is moderate, and its fracture line propagated to the lateral border of the LT. **c** The PLF in the 3-fragmentary L involved a substantial portion of the GT and LT as a whole piece. **d** The LT was separated from the PLF and the shaft fragment as the fourth fragment in the subtype 4-fragmentary GL

To eliminate the negative influences of initial displacement on the fracture morphology analysis, three-dimensional CTs (3D-CT) of both sides of the proximal femurs were reconstructed simultaneously. The fracture line at the basicervical region was designated the primary fracture line, and the coronally propagated fracture line affecting the GT was designated the secondary fracture line. The analysis of the reconstructed 3D images revealed that there were three consistent fragments: cephalocervical fragment, femoral shaft and posterolateral fragment from GT. A variable fragment (LT) was also frequently identified. Therefore, each case could be defined as either a 3-fragmentary or 4-fragmentary pattern. Then, the rationale of Shoda’s 3D-CT classification of the proximal femur was borrowed to further classify it into one of four subgroups [7]. The 3-fragmentary S was the first subgroup, where “S” referred to a small PLF fragment combined with cephalocervical and shaft fragments (Fig. 3a). The second subgroup was the 3-fragmentary M, in which a moderate PLF (indicated by “M”) coexisted with other two consistent fragments. Compared to the 3-fragmentary S, the secondary fracture line in the 3-fragmentary M propagated more medially and was in the vicinity of the lateral border of the intact LT (Fig. 3b). In the third subgroup, one large banana-like fragment consisting of the PLF and the LT as one unit occurred in the 3-fragmentary L (Fig. 3c). When the LT was also concomitantly fractured, there were four large fragments; thus, it was defined as the 4-fragmentary GL subtype (Fig. 3d).

**Fig. 4:**
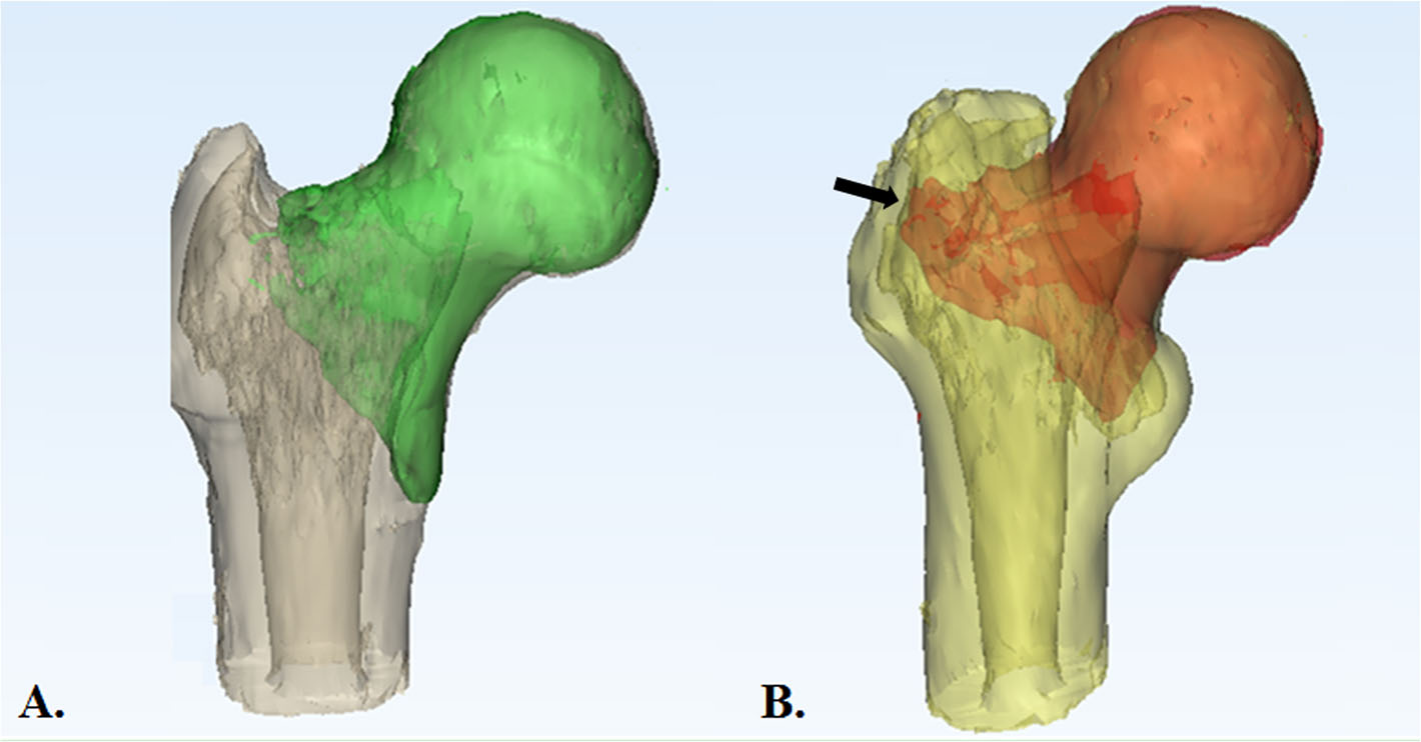
**a** The cephalocervical fragment was fused on the mirror image of the contralateral proximal femur. The primary fracture line of the injured side just crossed the piriform fossa. **b** The primary fracture line involved a small piece of the posteromedial wall of the GT

On the 3D-CT, the morphological feature of the primary fracture line was specifically studied [7]. As the GT of the injured limb was routinely comminuted and the normal spatial relationship between the main fragments was altered, we utilized the mirrored image of the contralateral uninjured proximal femur as a template for the injured hip to precisely evaluate the distribution primary fracture line. After the DICOM data were entered into Materialise 3-matic (Materialise NV Inc., Leuven, Belgium), the cephalocervical fragment of the TF was maximally matched to the mirrored 3D image of the contralateral femur. On the fused images, the relationship between the primary fracture line and the GT could be analyzed (Fig. 4).

**Fig. 5:**
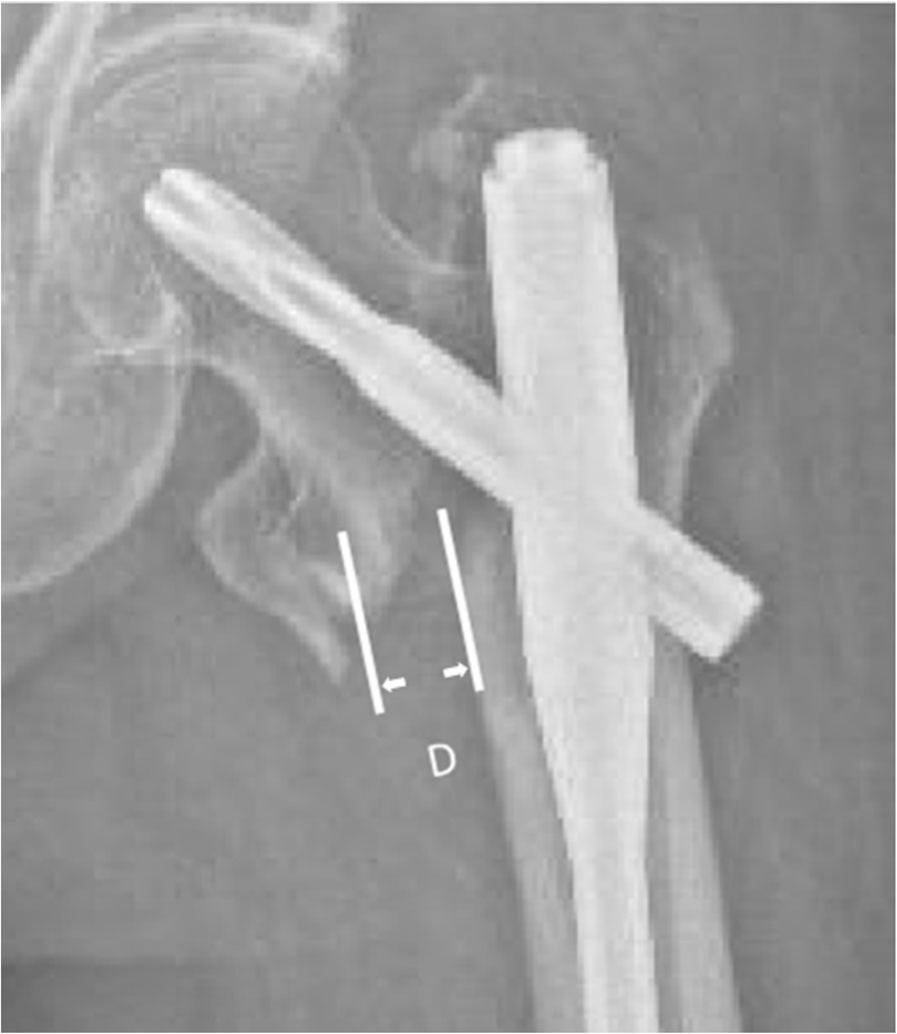
The tangential lines to the medial cortexes of the cephalocervical and shaft fragments are drawn. The distance between the two lines represents the width of the basicervical gap

Thirty-one patients were stabilized by proximal femoral nail anti-rotation (PFNA, Synthes Inc., Oberderf, Switzerland) and two younger patients by a TRIGEN INTERTAN nail (Smith–Nephew, Memphis, USA). The postoperative radiography was assessed by another fellow training surgeon who did not take part in the operation (H.G.Q). The width of the secondary displacement at the inferior part of the primary fracture line and the NSA were measured on the anteroposterior (AP) view (Fig. 5).

All statistical analyses were performed using Statistical Package for the Social Sciences (SPSS) (ver 23.0, International Business Machines, Inc. Armonk, New York, USA). Quantitative data were expressed as median and compared among groups by Student’s t-test or Mann-Whitney U test. All *P*-values were two-sided and a *P*-value   <  0.05 was considered to be statistically sign.

### Surgical technique

Under general anesthesia, the patient was positioned on the operation table in supination with consistent distraction of the injured limb, and the manual reduction of the fracture was performed. By persistent traction and an internal rotation maneuver, the limb length and rotational alignment were restored first. After the medial cortical continuity and acceptable NSA were confirmed on AP view of the fluoroscopic image, the anterior cortex reduction was checked on lateral view. If there was negative reduction of the anterior cortex, a small elevator was percutaneously inserted to reduce it. No attempt was made to reduce an isolated LT fragment or PLF from the GT.

When the reduction was satisfactory on both views, a PFNA/TRIGEN INTERTAN nail was inserted according to the manufacturer’s operation manual. After making a 5-cm incision proximal to the tip of the GT, the fasciae and muscle fibers of the gluteus medius were split. As the standard manipulation, the entry point of the nail was slightly lateral to the tip of the GT, and guide wire placement was followed by opening the femur, reaming the medullary canal and inserting a PFNA/TRIGEN INTERTAN nail. Frequently, “reverse wedge effect” occurred either during canal reaming or nail insertion.

**Fig. 6:**
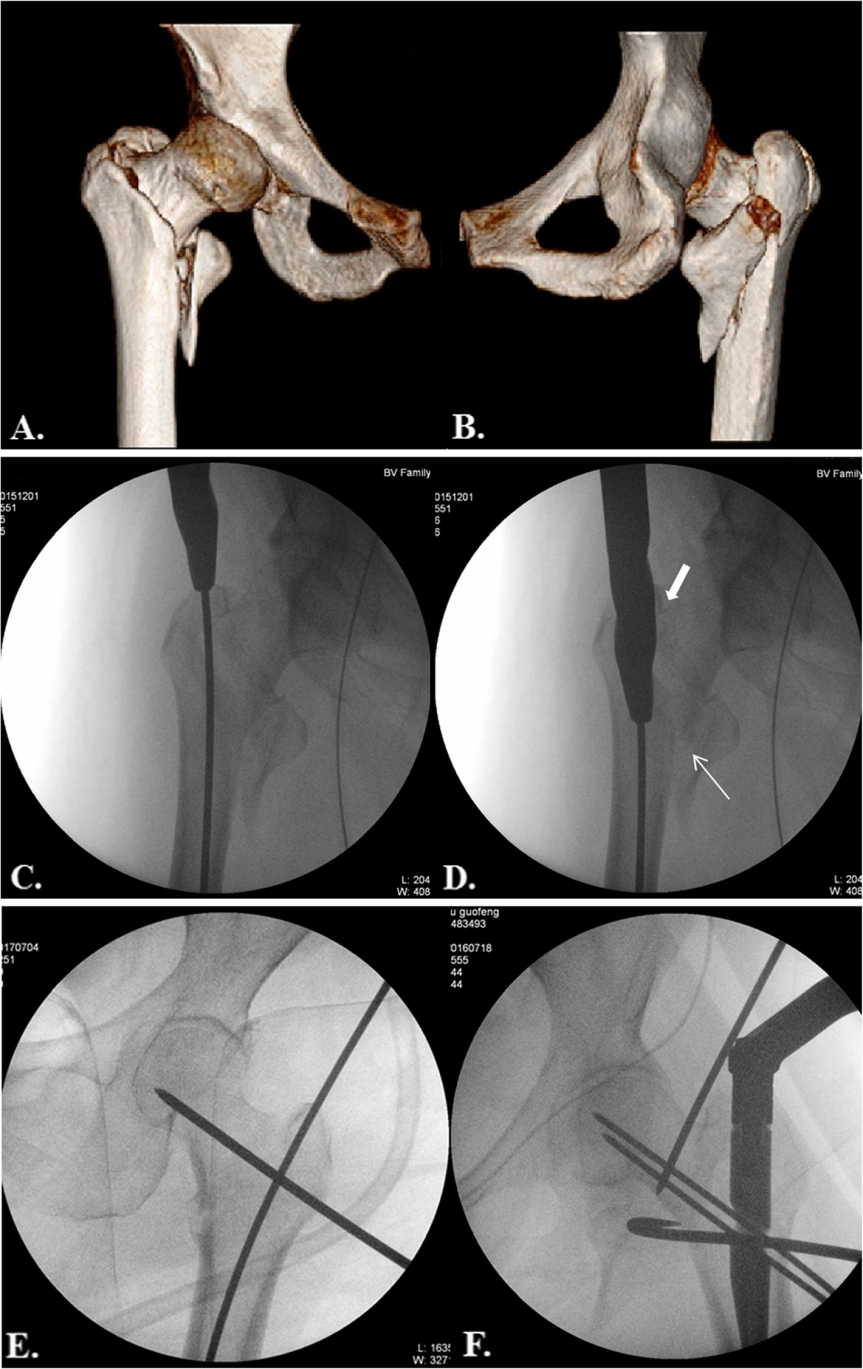
**a** and **b** Preoperative 3D image demonstrating a subtype 4-fragmentary GL pertrochanteric fracture with mild varus deformity. **c** and **d** The wide arrow indicates the impact between the reamer and the superolateral cortex of the cephalocervical fragment. The resultant basicervical gap is designated by a narrow arrow. **e** When the risk of reverse wedge effect was high, a 3.5-mm Kirschner wire (K-wire) was inserted to secure the reduction. **f** Occasionally, a single K-wire was not strong enough, and a bone hook was inserted to prevent inferior opening at the fracture line

Before 2017, we resorted to enlarging the entrance of the femoral canal or overdistracting the injured limb to overcome this secondary deformity. However, the design of the reamer made the over-reaming technique extremely difficult, and the overdistraction method generally failed. Later, we chose to secure the acquired initial reduction by inserting a 3.5-mm Kirschner wire (K-wire) before reaming and PFNA/TRIGEN INTERTAN nail insertion (Fig. 6e). Under image intensifier control, the K-wire was inserted along the axis of the femoral neck and closely underneath the anterior cortex. Then, the femoral canal was reamed, and the nail was inserted. A bone hook should sometimes be added to augment the maintenance of the primarily achieved reduction (Fig. 6f). We found that those methods were extremely effective and dramatically shortened the operation time if the risk of the “reverse wedge effect” could be predicted preoperatively.

## Results

A total of 33 patients met the inclusion criteria in the final analysis so that the overall incidence of the “reverse wedge effect” was 7.9%. The preoperative plain radiograph analysis revealed that all the 33 patients were basicervical TF variants while there were another 12 cases of this special fracture type did not present similar intraoperative complication. The patients included 12 males and 21 females, with a mean age of 72.5 years (33 to 96 years). The internal rotation of the cephalocervical fragment caused by reamer/IN impingement was the main underlying cause of this intraoperative complication. On the postoperative radiograph, this secondary displacement demonstrated an inferior gap at the primary fracture line and increased NSA (Fig. 2b). The AO/OTA classification scheme failed to properly categorize this special fracture type properly [8]: on the plain radiograph, the coronally oriented secondary fracture line made the measurement of the lateral wall thickness unreliable and generated great confusion in differentiating AO-31 A1 GT from A2. In contrast, the classification system we devised in this study depicted each case briefly and concisely.

The incidence of the four-fragment TF (4-fragmentary GL) was the highest (Table 1). In all four subgroups, the majority of the primary fracture lines transversed the center of the piriformis fossa (83.8%) (Fig. 4a). The remaining fracture lines marginally involved a small portion of the GT medial wall (Fig. 4b). Therefore, all the primary fracture lines originated extracapsularly.

**Table 1:** The 3-fragmentary and 4-fragmentary subgroups were compared with Mann-Whitney U tests

Subgroups	3-fragmentary	4-fragmentary	*p* value
Frequency	13	20	–
Median Displacement (mm)	8.2 (6.90, 8.85)	9.2 (8.45, 10.95)	0.068
Median NSA (degree)	136.0 (130.5, 138.5)	133.0 (132.0, 138.8)	0.624

A Mann-Whitney U test was run to determine if there were differences in magnitude of displacement and the NSA between 3-fragmentary and 4-fragmentary subgroups. Distributions of the secondary displacement and NSA for 3-fragmentary and 4-fragmentary subgroups were similar, as assessed by visual inspection. Median displacement for 3-fragmentary (8.20 mm) and 4-fragmentary (9.20 mm) was not statistically significantly different, U = 80.5, z = − 1.825, *p* = 0.068, using an exact sampling distribution for U. While, the median NSA for 3-fragmentary (136.0 degrees) and 4-fragmentary (133.0 degrees) was not statistically significantly different, U = 116.0, z = − 0.518, *p* = 0.624.

## Discussion

The purpose of the present study was to investigate the incidence, characteristics and causes of the “reverse wedge effect” during application of the IN in TF. In contrast to the previously reported “wedge effect”, which was defined as an intraoperative secondary displacement at the superior part of the pertrochanteric fracture line [6], we observed another IN-related intraoperative complication, which was demonstrated as an inferior opening at the already reduced primary fracture line. We also identified that all the cases occurred in basicervical TF variants combined with a disrupted GT. By reviewing intraoperative fluoroscopy, we noticed an impingement between the reamer/IN and the superolateral edge of the cephalocervical fragment, which caused this secondary displacement (Fig. 6). Because all the 414 reviewed cases were treated by PFNA/TRIGEN INTERTAN nailing but the incidence of the “reverse wedge effect” was nearly 7.97%, we believe that anatomic factors other than implantation should play a major role.

The basicervical fracture line is the most significant feature of this cohort of patients. Strictly, a basicervical fracture is defined as a 2-part fracture where the fracture line originates from the base of the femoral neck and exits above the lesser trochanter [9]. Despite the concomitant PLF or LT fracture, the primary fracture lines in this cohort of patients met this description exactly. There is little controversy about the fact that a basicervical fracture is an unstable fracture and prone to varus deformity [10]. Sliding screws were initially proposed as a treatment for these fractures [11, 12], but the recent trend favors IN fixation [9, 13]. Anatomically, the piriformis fossa is the transitional region between the hard cortex of the femoral neck and the relatively weak cancellous bone of the GT [14]. Our study demonstrated that the primary fracture lines just crossed the piriformis fossa center. This indicated that the superolateral corner of the cephalocervical fragment was primarily made from the hard cortex, which is hard enough to resist reaming and colliding with the reamer and PFNA/TRIGEN INTERTAN nail insertion. Eventually, the reamer or EFNA/TRIGEN INTERTAN nail would push the cephalocervical fragment, internally rotating and inferiorly displacing, resulting in the “reverse wedge effect”.

Notably, in this cohort, the PLF and LT fragment were the other two prominent anatomical features. As Cho’s study found that the incidence of the PLF in TF was as high as 88.4% [15], we observed a similar prevalence among all the 414 patients, and every “reverse wedge effect” case had a PLF. We believe that further classification and comparison would facilitate understanding and exploring the influences of those two fragments on “reverse wedge effect” formation and treatment/strategy design.

Although Van Embden stated that AO/OTA classification was more comprehensive and reliable compared to the Jensen-Evans classification [16], we found that it failed to cover this special fracture pattern. According to AO/OTA classification (2018 version), AO-31A1 and A2 were designated as pertrochanteric fractures in which the main fracture line propagated through the trochanters. Saarenpaa, Watson and other authors described basicervical TF variant as fracture at the base of femoral neck that is medial to the intertrochanteric line [9, 17, 18]. So basicervical TF variant could not be classified into pertrochanteric (AO - 31A1 and A2) or intertrochanteric fracture (AO - 31A3). In contrast, in 1949, even before the clinical application of computed tomography, Evans had proposed 3- and 4-fragmentary fracture patterns in his classification scheme [19]. Considering the complexity of the fracture morphology, a three-dimensional classification system originating from Evans’ would be more pragmatic [16]. The principle from Babhulkar and Shoda’s classifications of GT fracture [2, 7] could be incorporated to further classify basicervical TF variants according to the morphology of the PLF.

Although the sample size was not large enough to make statistical comparisons between the four subgroups, the measured displacements of “reverse wedge effect” and ultimate NSA were similar among them. Therefore, we could infer that the volume of the PLF minimally influenced the extent of the secondary displacement in this cohort of patients. From a mechanical view, it is the existence of the PLF contributes to the development of the “reverse wedge effect”.

When the GT region is intact, the guide wire could be constrained around the tip of the GT, and the trajectory of the reamer/IN was centralized into the femoral canal. However, an incompetent GT made the guide wire and reamer float at the start site. Under an image intensifier, we observed that the reamer was prone to skew medially, especially when attempting to over-ream the superolateral corner of the cephalocervical fragment (Fig. 6d). As a result, impaction between the cephalocervical fragment and the reamer/nail occurred. Since the volume of the PLF plays a relatively minor role, this fragment could be excluded during the management of the “reverse wedge effect”.

In this cohort of patients, the incidence of PLF was 100% and the LT disruption was 60.6%. To investigate the role of LT disruption in this operative complication, comparisons between 3- and 4-fragmentary subgroups in the magnitude of displacement and postoperative NSA were made and revealed no significant differences. We could conclude that the appearance of LT fragment contributed minimally to the occurence and degree of the “reverse wedge effect”. When preventing the reverse wedge effect in basicervical TF variant, the lesser trochanter fragment did not have to be reduced and temporarily stabilized.

It is unclear how this intraoperative complication negatively impacts the treatment outcome. The postoperative measurement demonstrated that the magnitude of the secondary displacement in the “reverse wedge effect” was significant. Zhang et al. advocated the “medial positive reduction” concept to validate a stable medial cortex mismatch, but they did not quantify the threshold of the acceptable diastasis at the medial cortex [20]. It is reasonable that an obvious opening at the primary fracture line represents instability in the fracture. Thereafter, postoperative excessive collapse would occur because of failure to restore cortex interdigitation. Early postoperative weight bearing had to be postponed. The postoperative radiograph demonstrated a mild tendency of valgus reduction in the majority of patients. Although mild valgus reduction is more preferred than varus reduction for allowing interfragment compression and reducing bone-implant stresses, Ciufo et al. recently showed that residual basicervical gapping was closely associated with fixation cutout [21]. Considering that the magnitude of the secondary displacement exceeded 4 mm, which was proposed by Baumgaertner as the lowest threshold for malreduction [22], we believe that this newly reported intraoperative complication is worth further investigation.

There were some limitations to the present study. First, this study was a retrospective analysis. Therefore, the robustness of the analysis is undetermined. Second, a comparison between the different trochanteric fracture patterns in the incidence rate of the “reverse wedge effect” was not carried out. However, we rigorously reviewed all the intraoperative fluoroscopy findings during the study period and did not find any “reverse wedge effect” in other fracture patterns. Thus, we believe that this complication is closely correlated with the nailing basicervical TF variant combined with the PLF. Third, the treatment outcomes of this cohort of patients were not evaluated for several reasons. The primary reason is that this cohort of patients was highly heterogeneous, and some very young patients were included, which made building an age- and sex-matched control group difficult.

## Conclusion

Our study reveals traumatic anatomic factors associated with the “reverse wedge effect” in cephalomedullary nailing in a group of trochanteric fractures. In contrast to the previously proposed “wedge effect”, this “reverse wedge effect” is characterized by an inferior opening at the primary fracture line in the trochanteric region and a mild valgus malreduction. This secondary displacement exclusively occurred during the reaming/nailing of an anatomically reduced trochanteric fracture featuring a basicervical variant type and a disrupted GT. Further analysis demonstrated that the basicervical fracture line is the major contributor to this intraoperative complication and that the impingement between the reamer/nail and the superolateral cortex tip of the cephalocervical fragment was the direct cause. Thus, the magnitude of the displacement is significant, and early postoperative weight bearing might be compromised. We advocate that this special intraoperative complication should be prevented in advance of reaming and nail insertion to optimize the final reduction quality.

## Data Availability

Yu Zhang will be contact to provide data and materials.

## Abbreviations

IN: Intramedullary nailing
GT: Greater trochanter
TF: Trochanteric fractures
CT: Computed tomography
NSA: Neck-shaft angle
PLF: Posterolateral fragment
LT: Lesser trochanter
PACS: Picture Archiving and Communication System
3D-CT: Three-dimensional CTs
PFNA: Proximal femoral nail anti-rotation
AP: Anteroposterior
K-wire: Kirschner wire
